# Association between Asthma and Lower Levels of Physical Activity: Results of a Population-Based Case–Control Study in Spain

**DOI:** 10.3390/jcm13020591

**Published:** 2024-01-19

**Authors:** Javier De-Miguel-Diez, Carlos Llamas-Saez, Teresa Saez Vaquero, Rodrigo Jiménez-García, Ana López-de-Andrés, David Carabantes-Alarcón, Francisco Carricondo, Barbara Romero-Gómez, Napoleón Pérez-Farinos

**Affiliations:** 1 Respiratory Care Department, Gregorio Marañón General University Hospital, 28007 Madrid, Spain; javier.miguel@salud.madrid.org; 2 Department of Public Health and Maternal & Child Health, Faculty of Medicine, Complutense University of Madrid, 28040 Madrid, Spain; carlolla@ucm.es (C.L.-S.); anailo04@ucm.es (A.L.-d.-A.); dcaraban@ucm.es (D.C.-A.); 3 Madrilenian Health Service, Ministry of Health, 28046 Madrid, Spain; mteresa.saezva@salud.madrid.org; 4 Department of Immunology, Laboratory of Neurobiology of Hearing (UCM 910915), Ophthalmology and Otorhinolaryngology, Faculty of Medicine, Complutense University of Madrid, 28040 Madrid, Spain; fjcarric@ucm.es (F.C.); brgomez@ucm.es (B.R.-G.); 5 Epi-PHAAN Research Group, School of Medicine, Universidad de Malaga, 29071 Malaga, Spain; napoleon.perez@uma.es

**Keywords:** asthma, physical activity, adults, sex differences, survey

## Abstract

(1) Background: Our aim was to determine changes in the prevalence of physical activity (PA) in adults with asthma between 2014 and 2020 in Spain, investigate sex differences and the effect of other variables on adherence to PA, and compare the prevalence of PA between individuals with and without asthma. (2) Methods: This study was a cross-sectional, population-based, matched, case–control study using European Health Interview Surveys for Spain (EHISS) for 2014 and 2020. (3) Results: We identified 1262 and 1103 patients with asthma in the 2014 and 2020 EHISS, respectively. The prevalence of PA remained stable (57.2% vs. 55.7%, respectively), while the percentage of persons who reported walking continuously for at least 2 days a week increased from 73.9% to 82.2% (*p* < 0.001). Male sex, younger age, better self-rated health, and lower body mass index (BMI) were significantly associated with greater PA. From 2014 to 2020, the number of walking days ≥2 increased by 64% (OR1.64 95%CI 1.34–2.00). Asthma was associated with less PA (OR0.87 95%CI 0.47–0.72) and a lower number of walking days ≥2 (OR0.84 95%0.72–0.97). (4) Conclusions: Walking frequency improved over time among people with asthma. Differences in PA were detected by age, sex, self-rated health status, and BMI. Asthma was associated with less LTPA and a lower number of walking days ≥2.

## 1. Introduction

Asthma is a common disease characterized by airflow limitation and respiratory symptoms associated with chronic airway and systemic inflammation, bronchial hyperreactivity, and exercise-induced bronchoconstriction. It is associated with a serious health burden, underscoring the importance of symptom management and disease control [1]. Thus, once the diagnosis of asthma has been made, treatment should begin to control symptoms and reduce the risk of exacerbation. Pharmacological stepwise therapy is the first line of treatment for asthma. Current treatment guidelines for this disease highlight management with inhaled corticosteroids alone or in combination with a long-acting beta-agonist [2,3,4]. Severe forms of asthma may require other pharmacological strategies and biological therapies and may even need hospitalization and ventilatory support [5,6,7]. Recent controversy has been raised regarding the use of ketamine in severe asthma exacerbations. However, a recent review does not support the use of this drug as so far, and a limited number of prospective studies have been published with large heterogeneity [5]. Biological therapies include monoclonal antibodies directed to key inflammatory cytokines involved in asthma pathogenesis [6]. The use of noninvasive ventilation is recommended for severe asthma as it may prevent the need for endotracheal intubation in selected patients; this is important since patients who have been intubated for severe asthma are at increased risk of death [7]. Nevertheless, optimal asthma control is complicated by costs, drug side effects, and a lack of adherence to prescribed medication. Poor adherence to prescribed medication necessitates more frequent emergency treatment, thus increasing health system costs [2,3,4].

Different non-pharmacological therapeutic approaches can also improve the symptoms of patients with asthma, but in many cases they are not applied [8]. Although their potential benefits have not been sufficiently studied [9,10], current guidelines recommend that patients with asthma undertake regular physical activity (PA) [11,12]. Engaging in leisure time PA (LTPA) has proven to be a cost-effective strategy, as it improves symptoms, asthma control, pulmonary function, health-related quality of life, and use of health services [9,13,14]. Thus, PA is an important outcome that should be assessed in patients with asthma [15].

Despite the benefits described, population-based studies have revealed that patients with asthma engage in less PA than those without asthma [16], probably owing to several barriers, including fear of exercise-induced bronchoconstriction [17], a transitory condition that occurs in 40% to 90% of people with asthma and causes airway narrowing during or after exercise [1,17,18]. Several pharmacologic approaches can be adopted to prevent this phenomenon, with bronchodilators representing the mainstay approach [19]. However, patients suffering from exercise-induced bronchoconstriction may limit or avoid exertion due to symptoms of shortness of breath, cough, wheezing, and chest tightness. This could have negative consequences for their health, since exercise avoidance may increase social isolation, and this can lead to obesity and poor health. In fact, exercise has paradoxically been shown to improve exercise-induced bronchoconstriction severity, pulmonary function, and airway inflammation in these patients. Early detection, diagnosis confirmed by the change in lung function during exercise, and appropriate treatment can improve their quality of life, allowing patients with asthma to perform PA without limitations [20].

Psychological issues and lack of motivation have been also reported as barriers to PA in patients with asthma. Applying behavior change techniques as part of a PA program may help overcome the mentioned barriers and facilitate PA in these patients [21].

Identifying changes in the PA of patients with asthma over time and associated factors make it imperative to conduct health promotion activities and lifestyle interventions in those at risk of low PA and, therefore, reduce health disparities. We aimed to determine whether the prevalence of PA in adults with asthma changed between 2014 and 2020 in Spain. We also investigated possible sex differences and the effect of other sociodemographic and health-related variables on adherence to PA among adults with asthma. Finally, we compared the prevalence of PA between individuals with asthma and age- and sex-matched individuals without this condition.

## 2. Materials and Methods

We conducted an observational study encompassing both cross-sectional and case–control designs. The data utilized for this research were sourced from the European Health Interview Survey for Spain (EHISS) corresponding to the years 2014 and 2020. These surveys are undertaken on a representative sample of the Spanish population comprising over 22,000 individuals aged ≥15 years residing in private households.

The recruitment periods spanned 12 months to account for seasonal variability, namely January to December 2014 for EHISS 2014 and July 2019 to June 2020 for EHISS 2020. Data collection was facilitated through face-to-face interviews with participants in their homes. Owing to the COVID-19 pandemic, data collection transitioned to telephone interviews between March and July 2020. Detailed information regarding the EHISS can be accessed online [22,23].

The EHISS uses a three-stage random sampling method to ensure nationwide and autonomous community representation. The final sampling level is the household, where, if multiple individuals aged ≥15 years reside, a single participant is randomly selected from the inhabitants.

Our study population comprised all individuals from both surveys aged 18 years and above. The analysis excluded participants who either did not respond or answered “I don’t know” to questions concerning asthma and the PA questions.

For the case–control study, each participant with asthma (case) from the study population was paired with a participant without asthma (control) matched by survey year, sex, and age. When multiple controls were available for a case, one was chosen at random.

### 2.1. Study Variables

All variables in this study are based on self-reported responses to questions in the 2014 and 2020 EHISS questionnaires. Supplementary Table S1 elucidates the survey questions, possible answers, and their categorization for the creation of variables.

Participants were classified as having asthma if they answered “Yes” to the question, “Have you been diagnosed with asthma by a physician?” Those who answered “No” were considered not to have asthma.

Our study outcomes were the frequency and type of PA during free time and the number of days the person interviewed walked for at least 10 consecutive minutes in a week.

The two questions utilized to quantify PA were as follows:

(1) What frequency of physical activity do you engage in during your free time? There were four potential responses: (i) I do not exercise. My leisure time is primarily sedentary. (ii) I engage in occasional physical or sports activities (e.g., walking, cycling, gardening, light gymnastics, recreational activities requiring minimal effort). (iii) I participate in physical activities several times a month (e.g., sports, gymnastics, running, swimming, cycling, team games). (iv) I undergo athletic or physical training multiple times a week. Based on these responses, the variable “leisure time physical activity” (LTPA) was created and classified into two categories: “None” for those who selected the first option, and “Occasional or frequent” for any of the other three choices.
(2) In a typical week, how many days do you walk for at least 10 consecutive minutes? Participants could respond within a range of zero to seven days. This led to the definition of the variable “Number of walking Days”, categorized as “None or one” and “Two or more”.

Table S1 also lists all the covariates analyzed, including sociodemographic characteristics, self-perceived health status, self-declared chronic illnesses diagnosed by a physician, alcohol consumption, current smoking habits, and body mass index (BMI), which was calculated using participant-provided weight and height data.

### 2.2. Statistical Analysis

Descriptive statistics for the study population included absolute and relative frequencies and means and standard deviations for quantitative variables. Proportions were compared using Fisher’s exact test for unpaired data and McNemar’s test for paired data. Mean differences were analyzed using the *t* test.

Multivariable logistic regression models were constructed to identify variables associated with LTPA and “number of walking days” among individuals with asthma. Two additional models evaluated the impact of self-reported asthma (outcome variable) on the PA variables, adjusting for other covariates and aiming to confirm the results of the matching method. Models were created following the guidelines suggested by Hosmer et al. [24] with four consecutive steps.

(1) Univariate analysis of each variable. (2) Selection of variables for multivariable model. We included variables that showed statistical significance in the univariate tests and those deemed scientifically relevant based on the reviewed literature or prior research. The inclusion of scientifically relevant variables, despite their statistical significance, ensured that the model was grounded in theoretical or empirical evidence. (3) Fit and refinement of the multivariable model. Post the initial model fitting, the significance of each variable included in the model was scrutinized with the Wald statistic, which tested the significance of individual coefficients in the model. Each variable’s coefficient in the multivariate model was compared with its coefficient in a univariate model. Variables that did not contribute meaningfully (as indicated by statistical tests and theoretical relevance) were removed. The model was then refitted, and this process of elimination and refitting continued until a robust model was achieved. The likelihood ratio test was used for comparing the fit of the new model against the previous versions, ensuring the retained variables were essential. (4) Final assessment of the model: Once a stable model was established, we assessed the linearity of the relationships and checked for interaction effects among variables.

It is important to remember that while this approach helps in understanding associations, it does not imply causation.

As a measure of association, the odds ratio (OR) with the 95% confidence interval (CI) was calculated.

All statistical analyses were conducted using STATA 14.0 (StataCorp LP., College Station, TX, USA).

### 2.3. Ethical Considerations

The anonymized EHISS datasets are freely accessible on the Ministry of Health’s website, obviating the need for ethics committee evaluation [25].

## 3. Results

The total number of individuals with asthma interviewed in EHISS 2014 and EHISS 2020 was 1262 and 1103, respectively. In both surveys, women represented approximately 60%. The mean age increased slightly from 52.7 to 54.1 years (*p* = 0.073). As shown in Table 1, almost half of the participants with asthma perceived their health as fair/poor/very poor. If we analyze the two surveys together, we find that the most frequently self-reported comorbidities were high blood pressure (32.6%), chronic obstructive pulmonary disease (24.5%), and mental disorders (23.9%). Regarding lifestyles, 24.2% were obese (BMI ≥ 30), 20.5% smoked, and 52.3% consumed alcohol. None of these variables changed in frequency from 2014 to 2020.

The prevalence of occasional or frequent LTPA remained stable, with values of 57.2% in EHISS2014 and 55.7% in EHISS2020 (*p* = 0.45), and the proportion of individuals with asthma who reported walking continuously for at least two days a week increased significantly from 73.9% to 82.2% (*p* < 0.001).

**Figure 1:**
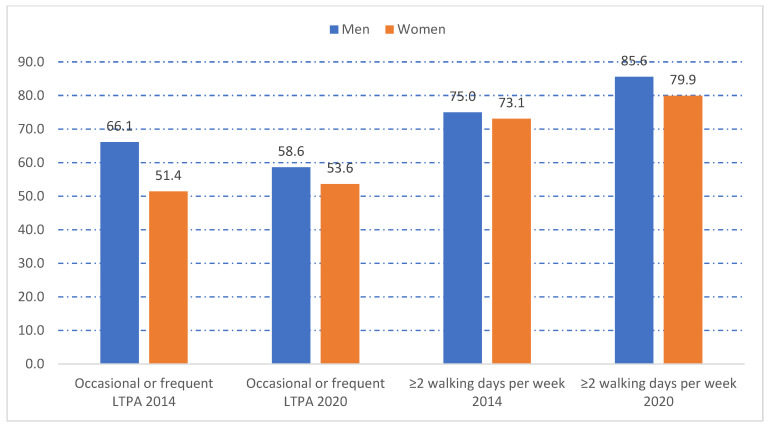
Frequency of occasional or frequent leisure time physical activity (LTPA) and number of walking days per week ≥2 according to sex among participants in the European Health Interview Surveys for Spain (EHISS) conducted in years 2014 and 2020, with self-reported asthma. LTPA: leisure time physical activity. All differences between men and women were statistically significant (*p* < 0.01).

**Figure 2:**
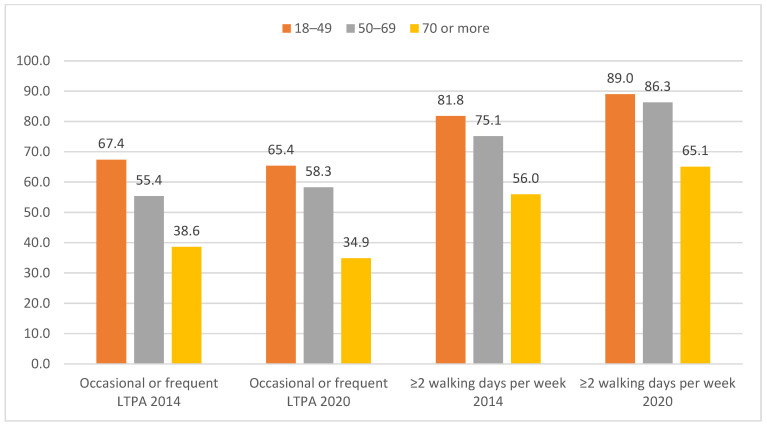
Frequency of occasional or frequent leisure time physical activity (LTPA) and number of walking days per week ≥2 according to age groups among participants in the European Health Interview Surveys for Spain (EHISS) conducted in the years 2014 and 2020, with self-reported asthma. LTPA: leisure time physical activity. Age was associated with occasional or frequent LTPA and ≥2 walking days per week in both surveys (*p* < 0.01).

Figure 1 shows the prevalence of occasional or frequent LTPA and the number of walking days ≥2 according to sex. Women with asthma reported less PA, measured with either of the two study variables, in both surveys. Analysis by age group (Figure 2) showed that both occasional and frequent LTPA and the number of walking days ≥2 decreased with age in 2014 and 2020.

Table 2 compares the prevalence of occasional or frequent LTPA and the number of walking days ≥2 between individuals with asthma and their age- and sex-matched controls according to sociodemographic variables. For all the categories of these covariables, individuals with asthma reported lower values for occasional or frequent LTPA and number of walking days ≥2 than controls. Among individuals with asthma, a higher educational level was associated with a higher prevalence of the two PA variables.

Table 3 shows the prevalence of occasional or frequent LTPA in individuals with asthma in the EHISS for 2014 and 2020 and their matched controls stratified by self-rated health, comorbidities, and lifestyles. Individuals with asthma had significantly lower prevalence values for occasional or frequent LTPA than the matched controls in all the covariables studied except self-rated health of very good/good and current smoking, where no difference was found. The lowest levels of occasional or frequent LTPA among the asthma population were observed in those who reported stroke (28.8%), heart disease (35.3%), and diabetes (37.2%). On the other hand, the highest prevalence was found among those who self-rated their health as very good/good (69.9%) followed by those with the lowest BMI (63%).

Table 4 shows the prevalence of “number of walking days ≥2” in individuals with asthma in the EHISS for 2014 and 2020 and their matched controls stratified by self-rated health, comorbidities, and lifestyles. The prevalence of “number of walking days ≥2” among individuals with asthma was lower than among controls without asthma for heart disease (57.7% vs. 63.1%; *p* < 0.01), stroke (47.9% vs. 54.9%; *p* < 0.01), cancer (66.6% vs. 72.3%; *p* < 0.01), mental disorders (59.6% vs. 61.8%; *p* = 0.017), high blood pressure (69.2% vs. 74.7%; *p* < 0.01), and BMI > 30 (68.7% vs. 76.6%; *p* < 0.01).

The results of the multivariable analysis to identify variables associated with occasional or frequent LTPA and the number of walking days ≥2 among participants with asthma are shown in Table 5. Male sex, younger age, better self-rated health status, and lower BMI were significantly associated with more PA measured using both study variables. Of the comorbidities studied, only heart disease was associated with less PA measured according to the two variables, and mental illness was associated with a lower number of walking days ≥2. Smoking at the time of the survey was associated with less LTPA. The analysis of changes between 2014 and 2020 revealed that the number of walking days ≥2 had increased by 64% (OR 1.64 95%CI 1.34–2.00).

The effect of having asthma, after adjusting for the remaining covariables, is shown in Table S2. Having asthma was associated with less LTPA (OR 0.87 95%CI 0.47–0.72) and a number of walking days ≥2 (OR 0.84 95%0.72–0.97).

## 4. Discussion

Our study revealed favorable trends in PA among patients with asthma, although these are still far from optimal. In fact, the prevalence of any type of LTPA among adult patients with asthma was low and did not change significantly over time. These findings indicate that a gap continues to exist between clinical guideline recommendations [7] and current practice, as previously reported for pharmacological treatment [26,27,28]. This may be a consequence of various barriers, both internal (lack of awareness, unfamiliarity, disagreement with the content of the guidelines, and lack of knowledge about their effectiveness) and external (patient-related or environmental factors) [29,30]. However, we demonstrated that the proportion of individuals with asthma who report walking continuously for at least two days a week was higher and increased significantly over time. The challenge that remains now is to overcome the heterogeneous and complex nature of this disease and achieve significant changes in participation in PA over time [21].

Our results are in line with previous reports that asthma patients undertake less PA than those without asthma [9,31,32] and that the level of PA in asthma is influenced by age and sex [9,33,34]. Regarding age, we observed that both LTPA and the number of walking days ≥2 decrease as age increases. Among the plausible biological mechanisms that could justify this association are the changes that affect the lungs in relation to age, which lead to an increase in breathing effort and are more marked in individuals with respiratory diseases. Furthermore, disease duration is more likely to be longer in older asthma patients, who consequently undergo a greater degree of airway remodeling [9,35]. Additionally, it has been reported that, in older asthma patients, the decrease in PA appears earlier in women than in men [36,37].

Previous studies have also reported lower PA in women than in men with asthma [38,39]. A possible explanation is that the consequences of this disease are more serious or have a more marked impact in women. In fact, among patients with asthma with a similar severity of airway obstruction, women tend to report poorer control [9].

Another factor associated with PA in our study was self-rated health status. Several studies have shown that an increase in PA improves quality of life in patients with asthma [40]. One of the reasons that could justify this association is that PA reduces the likelihood of asthma symptoms owing to cardiorespiratory adjustment to exercise, a decrease in BMI, improved self-esteem and mood, and less frequent symptoms of anxiety and depression [41]. In fact, PA and self-rated health have been shown to be related to good mental health in patients with asthma. Therefore, asthma patients who engage in PA and have a favorable self-rated health status present less psychological distress [2].

Our study also showed that a higher BMI was associated with reduced PA in asthma patients. The combination of obesity and reduced PA is associated with poor asthma control and a weaker response to treatment [42,43]. Therefore, it is necessary to take into account that obesity is associated with other chronic diseases, which, in turn, could be involved in lower PA levels [44].

Regarding comorbidity, we found that heart disease was associated with less PA measured according to the two variables and that mental illness was associated with a lower number of walking days ≥2. Comorbidities and decreased PA are especially relevant in the management of asthma because they have been shown to contribute to an increase in the likelihood of symptoms and the risk of exacerbation [45].

COPD was one of the most frequently self-reported comorbidities in patients with asthma in our study. The term “asthma–COPD overlap” (ACO) has been applied to the condition in which a person has a persistent airflow limitation with clinical features of both asthma and COPD. In the patients with asthma, the prevalence of ACO ranges between 11.1% and 61.0% [46]. Harada et al. found that the prevalence of ACO among patients who were previously diagnosed with asthma was 27.1%, a figure slightly higher than that found in our study [47]. Patients with ACO have more frequent and severe attacks, resulting in a greater number of hospitalizations, emergency visits, and higher health costs than patients with asthma and COPD [48]. Regarding PA, it has been demonstrated that more than three out of ten adults with ACO do not achieve the established recommendations. Therefore, it is recommended to implement programs that promote the importance and benefits of PA among patients with ACO, focusing especially on older adults and those who are obese [49].

Smoking was associated with less PA in our study. It has also been associated with poor asthma control and exacerbation of the disease [50]. Thus, it is important to ask patients about this harmful habit and encourage them to quit, as repeated attempts to quit smoking can reduce smoking rates significantly [51].

The strengths of this study include the utilization of a structured survey conducted during two different periods, thus allowing for comparison, as well as the large sample size assessed. However, several limitations must be considered. First, a main limitation of this work is the absence of comparative data in the same population between the two periods. The EHISS surveys are conducted every six years. Between the EHISS2014 and the EHISS2020, in the years 2016/17, the National Health Interview Survey of Spain was carried out with a similar methodology, but unfortunately, some of the variables used in the EHISS are collected with different questions, which means these surveys could not be merged for joint analysis [52]. Second, the last EHISS was carried out in 2020, the year the COVID-19 pandemic began; therefore, the lack of improvement in PA compared with the 2014 survey could have been due to social isolation during the pandemic. Third, in our investigation, we lack relevant clinical information, as it is not collected in the questionaries of the EHISS, such as which patients were being treated with inhaled corticosteroids, the severity of asthma, or the degree of control obtained. Fourth, it is well known that the presence of atopy may influence PA in terms of environmental exposure. Unfortunately, the EHISS surveys do not include atopy as one of the conditions inquired about. In any case, the EHISS surveys are conducted throughout the twelve months of the year to avoid the seasonal effect of environmental exposure on some diseases [22,23]. Fifth, population surveys lack many variables, such as those pointed out in the previous limitations, which are relevant and should be included in multivariable models in future research. In any case, the practice of PA is not usually recorded in clinical histories, and population surveys, despite their limitations, have been used to evaluate the practice of PA, from an epidemiological point of view, by various authors [2,3,31,32,39]. Sixth, as this is a cross-sectional study, it does not add information on whether the associations are causal and, if so, their significance. Seventh, as we used self-reported answers on diseases that were not confirmed with medical histories, the existence of social desirability or recall biases cannot be dismissed. Finally, the measurement of PA using questionnaires is another limitation, since it is not possible to quantify activity objectively.

## 5. Conclusions

In summary, although the proportion of individuals with asthma who report walking continuously for at least two days a week has increased significantly from 2014 to 2020, our study shows that the prevalence of any type of LTPA among adult patients with asthma is low and has not changed significantly over time. Male sex, younger age, better self-rated health, and lower BMI were significantly associated with more PA measured according to both study variables. Asthma was associated with less LTPA and a lower number of walking days ≥2 than in individuals who did not have asthma. Therefore, healthcare providers should identify the risk factors and clinical characteristics that are associated with reduced PA in patients with asthma and develop strategies to modify behavior and to increase PA in this population.

## Figures and Tables

**Table 1:** Distribution according to study variables of participants in the European Health Interview Surveys for Spain (EHISS) conducted in the years 2014 and 2020, with self-reported asthma.

Variable	Categories	EHISS 2014		EHISS 2020		
		n	%	n	%	*p*
Sex	Men	496	39.3	457	41.4	0.292
	Women	766	60.7	646	58.6	
Age (years)	Mean (SD)	52.7	(19.0)	54.1	(18.8)	<0.001
Age, year groups	18–49 years	599	47.5	492	44.6	0.364
	50–69 years	370	29.3	336	30.5	
	≥70 years	293	23.2	275	24.9	
Educational level	No studies/primary	702	55.6	586	53.1	0.221
	Secondary	209	16.6	212	19.2	
	High education	351	27.8	305	27.7	
Living with a partner	Yes	696	55.2	550	49.9	0.010
Self-rated health	Fair/poor/very poor	614	48.7	541	49.0	0.848
	Very good/good	648	51.3	562	51.0	
Diabetes	Yes	152	12	133	12.1	0.992
Heart diseases	Yes	168	13.3	163	14.8	0.305
Stroke	Yes	35	2.8	38	3.4	0.346
Cancer	Yes	67	5.3	70	6.3	0.281
COPD	Yes	327	25.9	253	22.9	0.094
High blood pressure	Yes	401	31.8	371	33.6	0.336
Mental illness	Yes	300	23.8	266	24.1	0.845
Alcohol consumption	Yes	677	53.6	559	50.7	0.150
Active smoking	Yes	277	21.9	209	18.9	0.072
Body mass index (kg/m^2^)	<25	512	40.6	424	38.4	0.405
	25–29.9	458	36.3	400	36.3	
	≥30	292	23.1	279	25.3	
Leisure time physical activity	None	540	42.8	489	44.3	0.450
	Occasional or frequent	722	57.2	614	55.7	
N° of walking days per week	None or one day	330	26.1	196	17.8	<0.001
	Two day or more	932	73.9	907	82.2	

Metal disease included “anxiety and/or depression”. COPD: chronic obstructive pulmonary disease. *p*-value for differences between EHISS 2014 and EHISS 2020 using Chi square and *t*-Student tests.

**Table 2:** Occasional or frequent leisure time physical activity and number of walking days per week ≥2 among subjects with asthma and sex/age-matched subjects without asthma participants in the European Health Interview Surveys for Spain (EHISS) conducted in the years 2014 and 2020 according to socio-demographic variables.

		Occasional or Frequent LTPA					Number of Walking Days per Week ≥2				
Variable	Categories	No Asthma		Asthma		*p*-Value	No Asthma		Asthma		*p*-Value
		n	%	n	%		n	%	n	%	
Sex ^a,c,d^	Men	648	68.1	596	62.5	<0.001	776	81.6	763	80.1	<0.001
	Women	810	57.3	740	52.4	<0.001	1123	79.5	1076	76.2	<0.001
Age groups ^a,b,c,d^	18–49 years	734	67.3	726	66.5	<0.001	912	83.6	928	85.1	<0.001
	50–69 years	462	65.5	401	56.8	<0.001	605	85.8	568	80.5	<0.001
	≥70 years	262	46.1	209	36.8	<0.001	382	67.3	343	60.4	<0.001
Educational level ^a,b,c,d^	No studies/primary	674	54.3	592	46	<0.001	947	76.3	917	71.2	<0.001
	Secondary	299	63.5	277	65.8	<0.001	400	84.9	357	84.8	0.958
	High education	485	74.4	467	71.2	<0.001	552	84.7	565	86.1	<0.001
Living with a partner	No	698	61.8	610	54.5	<0.001	905	80.1	858	76.7	<0.001
	Yes	760	61.6	726	58.3	<0.001	994	80.6	981	78.7	<0.001

LTPA: leisure time physical activity; *p* value for difference between participants with asthma and non-asthma age and sex matched controls. ^a^ Significant association between the variable and frequency of occasional or frequent LTPA among participants without asthma. ^b^ Significant association between the variable and number of walking days per week ≥2 days among participants without asthma. ^c^ Significant association between the variable and occasional or frequent LTPA among participants without asthma. ^d^ Significant association between the variable and number of walking days per week ≥2 days among participants with asthma.

**Table 3:** Occasional or frequent leisure time physical activity among subjects with asthma and sex/age-matched subjects without asthma participants in the European Health Interview Surveys for Spain (EHISS) conducted in the years 2014 and 2020 according to clinical variables and lifestyles.

Occasional or Frequent Leisure Time Physical Activity						
Variable	Categories	No Asthma		Asthma		*p* Value
		n	%	n	%	
Self-rated health ^a,b^	Fair/poor/very poor	288	40.9	490	42.4	<0.001
	Very good/good	1170	70.5	846	69.9	0.387
Diabetes ^a,b^	No	1365	63.2	1230	59.1	<0.001
	Yes	93	45.4	106	37.2	<0.001
Heart diseases ^a,b^	No	1352	63.8	1219	59.9	<0.001
	Yes	106	43.4	117	35.3	<0.001
Stroke ^a,b^	No	1436	62.1	1315	57.4	<0.001
	Yes	22	43.1	21	28.8	<0.001
Cancer ^a,b^	No	1411	62.2	1272	57.1	<0.001
	Yes	47	50.0	64	46.7	0.022
Mental illness ^a,b^	No	1299	63.8	1098	61.0	0.040
	Yes	159	48.3	238	42.0	<0.001
High blood pressure ^a,b^	No	1142	64.8	998	62.6	<0.001
	Yes	316	52.6	338	43.8	<0.001
Alcohol consumption ^a,b^	No	604	53.7	542	48.0	0.007
	Yes	854	68.9	794	64.2	<0.001
Active smoking ^a^	No	1155	63.3	1069	56.9	<0.001
	Yes	303	56.1	267	54.9	0.377
Body mass index ^a,b^	<25	742	65.7	590	63	<0.001
	25-29.9	518	61.8	509	59.3	<0.001
	≥30	198	49.9	237	41.5	<0.001

Mental illness included “anxiety and/or depression”. *p*-value for difference between participants with asthma and non-asthma age- and sex-matched controls. ^a^ Significant association between the variable and frequency of occasional or frequent LTPA among participants without asthma. ^b^ Significant association between the variable and occasional or frequent LTPA among participants without asthma.

**Table 4:** Number of walking days per week ≥2 among subjects with asthma and sex/age-matched subjects without asthma participants in the European Health Interview Surveys for Spain (EHISS) conducted in the years 2014 and 2020 according to clinical variables and lifestyles.

Number of Walking Days per Week ≥2						
Variable	Categories	No Asthma		Asthma		*p*-Value
		n	%	n	%	
Self-rated health ^a,b^	Fair/poor/very poor	477	67.8	794	68.7	0.347
	Very good/good	1422	85.7	1045	86.4	0.316
Diabetes ^a,b^	No	1762	81.1	1651	79.9	0.057
	Yes	137	66.8	188	66.0	0.111
Heart diseases ^a,b^	No	1745	82.3	1648	81.0	0.151
	Yes	154	63.1	191	57.7	<0.001
Stroke ^a,b^	No	1871	80.9	1804	78.7	0.035
	Yes	28	54.9	35	47.9	<0.001
Cancer ^a,b^	No	1831	80.7	1748	78.5	<0.001
	Yes	68	72.3	91	66.4	0.049
Mental illness ^a,b^	No	1661	81.6	1461	81.2	0.745
	Yes	197	61.8	320	59.6	0.017
High blood pressure ^a,b^	No	1450	82.2	1305	81.9	0.420
	Yes	449	74.7	534	69.2	<0.001
Alcohol consumption ^a,b^	No	866	77	816	72.3	<0.001
	Yes	1033	83.3	1023	82.8	0.721
Active smoking	No	1467	80.4	1449	77.1	<0.001
	Yes	468	60.4	486	59.8	0.921
Body mass index ^b^	<25	920	81.5	759	81.1	0.821
	25–29.9	601	81.4	467	73.1	0.855
	≥30	304	76.6	392	68.7	<0.001

Mental illness included “anxiety and/or depression”. *p*-value for difference between participants with asthma and non-asthma age- and sex-matched controls. ^a^ Significant association between the variable and number of walking days per week ≥2 days among participants without asthma. ^b^ Significant association between the variable and number of walking days per week ≥2 days among participants with asthma.

**Table 5:** Variables associated with occasional or frequent leisure time physical activity and number of walking days per week ≥2 among participants with asthma. Results of multivariable logistic regression analysis.

		Occasional or Frequent LTPA	Number of Walking Days per Week ≥2
		OR (95% CI)	OR (95% CI)
Sex	Women	1	1
	Men	1.46 (1.22–1.76)	1.21 (1.01–1.46)
Age groups	≥75 years	1	1
	50–69 years	1.70 (1.32–2.20)	2.18 (1.66–2.86)
	18–49 years	1.61 (1.22–2.12)	1.93 (1.45–2.57)
Educational level	No studies/primary	1	NIFM
	Secondary	1.44 (1.11–1.86)	NIFM
	High education	1.61 (1.27–2.04)	NIFM
Self-rated health	Fair/poor/very poor	1	1
	Very good/good	1.97 (1.63–2.40)	1.55 (1.20–1.99)
Heart diseases	No	1	1
	Yes	0.71 (0.54–0.93)	0.64 (0.48–0.85)
Mental disorder	No	NIFM	1
	Yes	NIFM	0.74 (0.58–0.95)
Active smoking	No	1	NIFM
	Yes	0.67 (0.54–0.84)	NIFM
Body mass index	≥30	1	1
	25–29.9	1.73 (1.38–2.18)	1.64 (1.27–2.14)
	<25	1.83 (1.45–2.31)	1.48 (1.14–1.92)
YEAR	2014	1	1
	2020	0.94 (0.80–1.11)	1.64 (1.34–2.00)

LTPA: leisure time physical activity. Mental illness included “anxiety and/or depression”. OR: odds ratios. CI: confidence interval. NIFM: not included in final the model.

## Data Availability

The anonymized EHISS datasets are freely accessible and can be downloaded by anyone on the Ministry of Health’s website. https://www.sanidad.gob.es/estadEstudios/estadisticas/EncuestaEuropea/home.htm (accessed on 8 October 2023). All other relevant data are included in the paper.

## Supplementary Materials

The following supporting information can be downloaded at: https://www.mdpi.com/article/10.3390/jcm13020591/s1, Table S1: Definition of variables according to the questions included in the European Health Interview Surveys in Spain conducted in years 2014 and 2020; Table S2: Variables associated with occasional or frequent leisure time physical activity and number of walking days per week ≥2 among participants in the European Health Interview Surveys in Spain conducted in the years 2014 and 2020. Results of multivariable logistic regression analysis.
