# Supplementary material for: Association between Asthma and Lower Levels of Physical Activity: Results of a Population-Based Case–Control Study in Spain
Source: J Clin Med. 2024 Jan 19;13(2):591. doi: 10.3390/jcm13020591 (PMC10816609; doi:10.3390/jcm13020591)
Supplement: Supplementary file 1 [file jcm-13-00591-s001.zip › jcm-2813487-supplementary.pdf]

Table S1. Definition of variables according to the questions included in the European Health Interview Surveys in Spain conducted in the years 2014 and 2020.

Questions	Description and answer	Variables name	Categories
Have you been diagnosed with asthma by a physician?	1.Yes<br>2.No	Asthma	1. Case<br>2. Control
What type of physical activity do you engage in during your free time?	1. I do not exercise. My leisure time is primarily sedentary.<br>2. I engage in occasional physical or sports activities (e.g., walking, cycling, gardening, light gymnastics, recreational activities requiring minimal effort, etc.).<br>3. I participate in physical activities several times a month (e.g., sports, gymnastics, running, swimming, cycling, team games, etc.).<br>4. I undergo athletic or physical training multiple times a week.	Leisure time physical activity	1 . None Option 1<br>2. Occasional or frequent Options 2, 3 and 4
In a typical week, how many days do you walk for at least 10 consecutive minutes?	Number of days (0 to 7)	Number of days of walking per week	1. None or one day<br>2. Two or more days
Which is your sex?	1. Man<br>2. Women	Sex	1. Man<br>2. Women
How old are you?	Age in years	Age groups	1. 18-49<br>2. 50-69<br>3. ≥70
What level of education have you completed?	1. Does not know how to read or write<br>2. Incomplete primary education<br>3. Complete primary education<br>4. First stage of Secondary Education, with or without a qualification<br>5. Elementary Spanish Upper Secondary Education	Educational level	1. No studies/Primary: Options 1 to 3<br>2. Secondary: Options 4 to 8<br>3. High education: Options 9 and 10

	6. Upper secondary education<br>7. Intermediate vocational training or equivalent<br>8. Advanced vocational training or equivalent<br>9. University studies or equivalent<br>10. Over university (master, PhD….)		
What is your marital status?	1. Single<br>2. Married<br>3. Widower<br>4. Separated<br>5. Divorced	Living with a partner	1. Yes: Option 2<br>2. Nor: options 1, 3, 4 and 5

Table S1. Definition of variables according to the questions included in the European Health Interview Surveys in Spain conducted in the years 2014 and 2020. (Continued.)

Questions	Description and answer	Variables	Categories
In the past twelve month, how is your perception of your general health status?	1. Very good<br>2. Good<br>3. Fair<br>4. Bad<br>5. Very bad	Self-rated health	1. Very good/good: Options 1 and 2<br>2. Fair/poor/very poor: Options 3 to 5
Have you been diagnosed with diabetes by a physician?	1.Yes<br>2.No	Diabetes	1. Yes<br>2. No
Have you been diagnosed with heart diseases (heart failure or coronary disease by a physician?	1.Yes<br>2.No	Heart diseases	1. Yes<br>2. No
Have you been diagnosed with stroke by a physician?	1.Yes<br>2.No	Stroke	1. Yes<br>2. No
Have you been diagnosed with cancer by a physician?	1.Yes<br>2.No	Cancer	1. Yes<br>2. No
Have you been diagnosed with COPD by a physician?	1.Yes<br>2.No	COPD	1. Yes<br>2. No
Have you been diagnosed with high blood pressure by a physician?	1.Yes<br>2.No	High blood pressure	1. Yes<br>2. No

Have you been diagnosed with anxiety or depression by a physician?	1.Yes<br>2.No	Mental disease	1. Yes<br>2. No
During the past 12 months, how often have you had alcoholic beverages of any kind (i.e. beer, wine, spirits, distilled and mixed drinks, or other alcoholic beverages)?	1. Daily or almost daily<br>2. 5-6 days per week<br>3. 3-4 days per week<br>4. 1-2 days per week<br>5. 2-3 days in a month<br>6. Once a month<br>7. Less than once a month<br>8. Not in the last 12 months, have I stopped drinking<br>9. Never or just a few sips to taste it throughout life	Alcohol consumption	1. Yes: Options 1 to 6<br>2. No: Option 7 to 9
Could you tell me if you smoke?	1. Yes, I smoke daily<br>2. Yes, I smoke, but not daily<br>3. I don't currently smoke but have smoked before<br>4. I neither smoke nor have I ever smoked regularly	Active smoking	1. Yes: Options 1 and 2<br>2. No: Options 3 and 4
1. Could you tell me how tall you are, approximately, without shoes?<br>2. Could you tell me your weight, approximately, without shoes and clothes?	Body mass index is calculated with the formulae:<br>Weight in kg/ (Height in meters)^2	Body mass index	1. <25<br>2, 25-29.9<br>3. ≥30

COPD: chronic obstructive pulmonary disease.

Table S2. Variables associated with occasional or frequent leisure time physical activity and number of walking days per week ≥2 among participants in the European Health Interview Surveys in Spain conducted in the years 2014 and 2020. Results of multivariable logistic regression analysis.

		**Occasional or frequent LTPA**	**Number of walking days per week ⩾2**
		OR (95% CI)	OR (95% CI)
Gender	Women	1	1
	Men	1.54(1.35-1.75)	1.13(0.97-1.32)
Age groups	≥75 years	1	1
	50-69 years	1.91(1.60-2.28)	2.70(2.22-3.29)
	18-49 years	1.81(1.50-2.17)	2.61(2.12-3.21)
Educational level	No studies/primary	1	1
	Secondary	1.32(1.11-1.57)	1.35(1.08-1.69)
	High education	1.79(1.52-2.10)	1.35(1.10-1.65)
Heart diseases	No	-	1
	Yes	0.67(0.55-0.82)	NIFM
Stroke	No	1	1
	Yes	0.65(0.44-0.98)	NIFM
Mental disorder	No	1	1
	Yes	0.70(0.60-0.83)	NIFM
Diabetes	No	1	1
	Yes	0.78(0.63-0.96)	0.80(0.62-0.97)
Active smoking	No	1	-
	Yes	0.60(0.51-0.70)	0.79(0.65-0.95)
Body mass index	≥30	1	1
	25-29.9	1.64(1.39-1.94)	1.50(1.24-1.82)
	<25	1.77(1.39-1.94)	1.41(1.16-1.71)
Year	2014	1	1
	2020	0.95(0.84-1.08)	1.86(1.60-2.16)
Asthma	No	1	1
	Yes	0.87(0.47-0.72)	0.84 (0.72-0.97)

LTPA: leisure time physical activity; OR: odds ratios; CI: confidence interval; NIFM: not included in final the model.
